# Processing Complex Events in Fog-Based Internet of Things Systems for Smart Agriculture

**DOI:** 10.3390/s21217226

**Published:** 2021-10-30

**Authors:** Sandy F. da Costa Bezerra, Airton S. M. Filho, Flavia C. Delicato, Atslands R. da Rocha

**Affiliations:** 1 Postgraduate Program of Teleinformatics Engineering, Federal University of Ceara, Fortaleza 60455-970, Brazil; sandycosta@alu.ufc.br; 2 Departament of Teleinformatics Engineering, Federal University of Ceara, Fortaleza 60455-970, Brazil; airtonfilho@alu.ufc.br; 3 Institute of Computing, Fluminense Federal University, Niteroi 24210-310, Brazil; fdelicato@ic.uff.br

**Keywords:** Internet of Things, Fog computing, complex event processing

## Abstract

The recent growth of the Internet of Things’ services and applications has increased data processing and storage requirements. The Edge computing concept aims to leverage the processing capabilities of the IoT and other devices placed at the edge of the network. One embodiment of this paradigm is Fog computing, which provides an intermediate and often hierarchical processing tier between the data sources and the remote Cloud. Among the major benefits of this concept, the end-to-end latency can be decreased, thus favoring time-sensitive applications. Moreover, the data traffic at the network core and the Cloud computing workload can be reduced. Combining the Fog computing paradigm with Complex Event Processing (CEP) and data fusion techniques has excellent potential for generating valuable knowledge and aiding decision-making processes in the Internet of Things’ systems. In this context, we propose a multi-tier complex event processing approach (sensor node, Fog, and Cloud) that promotes fast decision making and is based on information with 98% accuracy. The experiments show a reduction of 77% in the average time of sending messages in the network. In addition, we achieved a reduction of 82% in data traffic.

## 1. Introduction

According to [1], the Internet of Things (IoT) environment is composed of physical and virtual entities where physical entities turn into virtual things inside a cyber-world. These things are embedded with different abilities such as sensing, analyzing, processing, and self-management capacities. By adopting interoperable communication protocols, these smart things should have unique identities and virtual personalities. The recent growth of Internet services and applications has contributed to increasing data processing and storage requirements. Such requirements are diverse in terms of the required resources by different applications, thus calling for customized solutions.

Edge computing recently emerged to overcome some drawbacks of using Cloud computing as a back-end platform for IoT. Such drawbacks include its unpredictable latency, lack of location awareness and user mobility [2]. The Edge computing concept aims to leverage the processing capabilities of IoT devices by using gateways, base stations, and other Edge devices, providing an intermediate tier between the end-devices (data producers/consumers) and the remote Cloud. The presence of Edge devices enables the local processing of requests, reducing the Cloud computing workload and application response time.

The Edge computing paradigm has been applied to several emerging scenarios and adopted by IoT applications that are time-critical and have sensitive data (in terms of privacy). One embodiment of this paradigm is Fog computing. Fog computing denotes a decentralized computing infrastructure consisting of processing nodes placed anywhere on the continuum between the end-devices and the Cloud. According to [3], Fog is a “cloud closest to the ground”. It extends the traditional Cloud computing architecture to the edge of the network, enabling the processing and analyzing of some data at that tier. The Fog architecture is generally composed of at least three tiers. The low tier consists of IoT devices that collect, pre-process and forward data to the Fog nodes. The middle tier comprises Fog nodes (with a higher processing power than the IoT devices) that perform data processing, decision making, and actuation. The Cloud stores information and processes data that Fog nodes did not have enough computing resources to process in the upper tier.

Fog computing bridges the gap between the Cloud and end-devices (e.g., IoT nodes) by enabling computing, storage, networking, and data management on network nodes within the close vicinity of IoT devices. Consequently, computation, storage, networking, decision making, and data management occur not only in the Cloud but also along the IoT-to-Cloud path as data traverses to the Cloud (preferably close to the IoT devices) [4]. One of the main differences between Cloud and Fog computing concerns the scale of hardware components associated with these computing paradigms. Cloud computing provides highly available computing resources at relatively high power consumption, whereas Fog computing provides moderate availability of computing resources at lower power consumption [5].

To perform raw data processing in the Fog and Cloud tier some techniques can be applied aiming to extract meaningful information, thus generating knowledge. In addition, other techniques are often used to perform pre-processing functions, with different goals, such as spurious data filtering and dimensional reduction. Data pre-processing techniques can help improve the quality of the data produced while reducing the use of resources in processing tasks. A widely adopted technique in both the steps of sensor data pre-processing and processing is data fusion. Data fusion [6] can be understood as a process of aggregating and combining data from multiple sources to prevent redundancy and reduce the response time. It can also improve data accuracy by using data from various sources to compose complete information. In addition to data fusion, complex event processing (CEP) techniques also contribute to improving the information generated by sensors. CEP goes beyond simple data query and transformation and aims to detect patterns in the data using filtering techniques, correlation, and creating new, composite events from simple ones.

In this context, this work proposes a multi-tier approach for complex event processing in Fog-based IoT systems. Our approach aims to process events based on sensing data with low response time and high accuracy. One of the goals of our proposal is to reduce the number of messages exchanged between the sensed area and the Cloud. The main idea is to identify a simple event that triggers the complex event processing, aiming to identify the nodes related to the event and extract the characteristics of the sensed location. In this way, information is processed in different tiers, according to the capacity of each device, optimizing the use of computational resources and decreasing the average response time, both between the sensor and the Fog tier and between the Fog tier and the Cloud. The data are collected, managed, and processed according to the application requirements and in a decentralized way. Different tiers are responsible for the collection, analysis, decision making, and support of IoT systems. Aiming to harness the intrinsic capabilities of the Fog computing environment, this approach also explores the typical concept of the geolocation Fog enhanced with an information context to consolidate the data in the Fog nodes. Sensor nodes are selected and orchestrated using their distance to the Fog node and the type of sensed data. So, our geographical distributed and context-aware approach can help to improve low latency and consistent data access.

The main contribution of this work is the proposal of a hierarchical approach of a complex event processing (CEP) mechanism aiming to (i) reduce data exchange and response time and (ii) increase information accuracy to make timely decisions and support a complete data processing workflow for smart agriculture applications.

Thus, among the advantages of our CEP approach in three tiers, we can cite:

- The CEP mechanisms distributed in the network are capable of load balancing with less processing power, generating complex events, and processing them in tiers with greater processing power;
- A new CEP mechanism can be implemented in the event of an increase in data volume, changing only the tier of the hierarchy in question;
- Network traffic to transfer information between tiers can be significantly reduced, sending only the data necessary for processing events at the appropriate tiers.

We simulated scenarios in precision agriculture to evaluate the proposed approach. It was tailored to the requirements of IoT applications from the smart agriculture domain. The experiments show a reduction of 77% in the average time of sending messages in the network and 82% of improvement in the throughput using the proposed approach compared to systems without our proposal. Additionally, tests were conducted to validate the proposed complex event processing engine.

This paper is organized as follows. Section 2 presents and discusses the related work. The proposed approach is presented in Section 3, and Section 4 covers the materials and methods. The results are discussed in Section 5. Finally, Section 6 concludes the paper.

## 2. Related Work

Managing data acquired by a vast array of heterogeneous sensors remains as complex as the knowledge needed to make a decision on the practices of an agricultural process. This is mainly due to the fact that processing is required in the various temporal and spatial dimensions of the acquired data. This task implies that data should become available in a suitable time window and format to be viewed and stored, but also to feed decision-making support systems. Managing data and extracting timely information is also quickly becoming a key issue. In this context, the authors in [7] proposed a universal CEP mechanism for IoT monitoring using Edge computing. In addition, a formalized hierarchical complex event model is created, including raw, simple, and complex events, to reduce the complexity of event modeling. The model uses complex time and space semantics to define flexible events through algorithms. The authors in [8] use a hybrid approach, in which two data processing tiers, the Edge-tier and Cloud-tier, work together to provide effective IoT data analysis. Specifically, raw data are locally collected at IoT devices. Features are extracted by performing data fusion techniques in devices with greater processing power and then sending the result to the Cloud.

The work described in [9] addresses the hierarchical data fusion in IoT networks that contain Edge devices, network and communication units, and Cloud platforms. Different data sources are combined at each tier to produce timely and accurate results. The work presented in [10] proposes utilizing complex event processing and a hierarchical distributed architecture for enabling data fusion at various levels. To this purpose, their paper introduces complex event processing (CEP) as a potential way of implementing sensor data fusion in distributed IoT systems. Aiming to leverage local processing capabilities wherever possible, or off-load tasks to Edge/Cloud computing otherwise—thereby paving the way for a multi-layered, hierarchical data fusion approach, aiming to reduce the network’s response time and amounts of transferred data. Another work dealing with a large amount of data is [11], where a new computing paradigm was proposed, designed to process Big Data in a collaborative Edge (CEE) environment. The work proposed the fusion of geographically distributed data, creating shared virtual visualizations of the data exposed to the end-users through interfaces predefined by the data owners.

In [12], the authors proposed and analyzed a CEP-based Fog architecture for real-time IoT applications that uses a publish–subscribe protocol. A testbed was developed with low-cost and local resources to verify the suitability of CEP-engines to low-cost computing resources. To assess the performance, they analyzed the effectiveness and cost of the proposal in terms of latency and resource usage, respectively. In [13], a weighted cost model is proposed to minimize IoT applications’ execution time and energy consumption in a computing environment with multiple IoT devices, multiple Fog/Edge servers, and Cloud servers. In addition, a new application placement technique based on the Memetic Algorithm is proposed to make batch application placement decisions for concurrent IoT applications. Due to the heterogeneity of IoT applications, they also proposed a lightweight pre-scheduling algorithm to maximize the number of parallel tasks for concurrent execution.

In [14], the authors proposed a scalable network architecture for monitoring and controlling agriculture in rural areas. Compared to the existing IoT-based farming solutions, the proposed solution reduces network latency up to a certain extent. In that paper, a cross-layer-based channel access and routing solution for sensing and actuating were proposed. The work of [15] used innovative platform technology to be applied to the Cloud agriculture platform. Cloud integration can be applied to large-area data collection and analysis, allowing farmland with limited network information resources to be integrated and automated. Those improvements, including agricultural monitoring automation, pest management image analysis, and monitoring can also be used to solve the predicament of large-area automation construction.

More specifically on precision agriculture, the authors of [16] proposed a framework designed to provide a complete farming ecosystem. The framework facilitates the simulation of custom farming scenarios for users, specifically to identify sensor placement, coverage area, line-of-sight deployment, data gathering through a relay mechanism or airborne systems, mobility models for mobile nodes, energy models for on-ground sensors and airborne vehicles, and back-end computing support using Fog computing paradigm. In [17], the authors proposed a multi-criterion-based resource allocation policy with resource reservation to minimize overall delay, processing time, and service level agreement (SLA) violations. This process considers Fog computing-related characteristics, such as device heterogeneity, resource constraints, and mobility, as well as dynamic changes in user requirements. The authors employed multiple objective functions to find appropriate resources for executing time-sensitive tasks in the Fog environment.

The work of [18] was a review to identify all relevant research on new computing paradigms with smart agriculture. They also proposed a new architecture model with the combinations of Cloud–Fog–Edge. Furthermore, the authors analyzed the agricultural application domains, research approaches, and the applications. Moreover, the survey discussed the components used in the architecture models and briefly explores the communication protocols used to interact from one layer to another.

All the studies mentioned above have contributed to improving Fog computing and CEP performance in different ways. However, their event model has been abstractly defined, some without hierarchical or specific description mechanisms. Therefore, focusing on the event model, data fusion, and information quality remain the unsolved technical issues our CEP approach currently addresses. Furthermore, some experiments were performed to evaluate our approach performance regarding data traffic, decision quality, and response time.

In our work, the fusion classification, as described in [19], based on the data type (redundant, cooperative, and complementary) is used in conjunction with the classification based on abstraction tiers (low, medium, and high). In addition, the proposal includes the integration of the CEP technique in the hierarchy of tiers. The CEP technique detects a significant number of events with low latency [20]. Our approach is described in Section 3. Advantages of the proposed CEP approach on three tiers can be outlined as follows:

- The CEP mechanisms distributed in the network can improve the information quality by adding information to the observed data;
- A new CEP mechanism can be implemented in the event of an increase in the volume of data, changing only the hierarchy tier in question;
- The response time of transferring information between tiers can be significantly reduced.

Table 1 lists the main contributions and differences between the works discussed in this section.

## 3. Multi-Tier Complex Event Processing Approach

**Figure 1:**
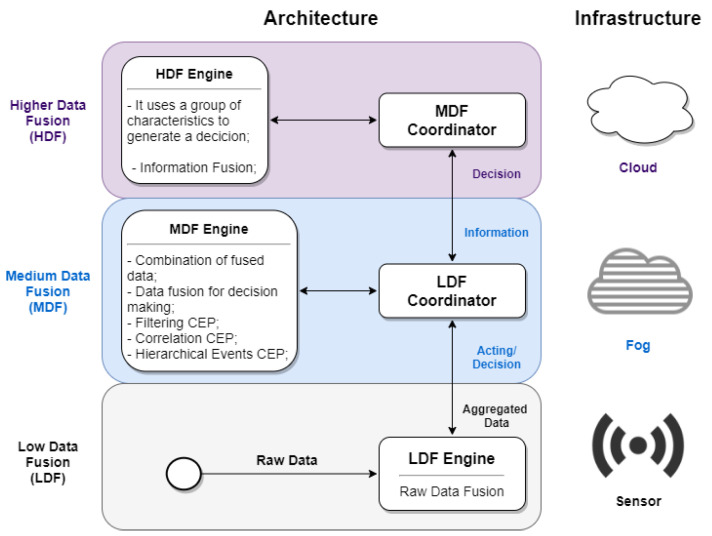
Conceptual architecture for multi-tier hierarchical CEP approach.

Modern IoT systems tend to go beyond the notion of “bottom–up” monitoring. Thus, sensing devices are not only in charge of collecting raw data and transferring it over the network. They also implemented a “descendant” feedback communication between the network tiers (e.g., for decision making based on data). The proposed approach uses coordination functionality as part of this two-way communication responsible for communication between the tiers described. More specifically, coordination is a two-way functionality. On the one hand, the Fog device receives data from lower-tier devices, collecting and dispatching to processors and data fusion engines at the upper tier if necessary. On the other hand, the Fog device sends the new processing rules to the lower tiers according to the changes observed in the data behavior. Thus, the proposed conceptual approach comprises the implementation and execution tiers of complex event processing in devices. Figure 1 describes the separation conceptual of tasks at all three tiers. These tasks have dedicated instances of data fusion (DF) mechanisms: low tier (LDF), medium tier (MDF), and high tier (HDF) data fusion [9]. The two top tiers include coordination components (LDF and MDF coordinators), responsible for bidirectional communication between the lower and upper-tier nodes and managing requests from the lower-tier nodes.

The architecture of our proposal (Figure 1) is composed of three different tiers which process simple and complex events from several different sources. The low-tier devices (IoT devices) collect data from the real world (for example, soil moisture and temperature). A relatively limited fusion technique (a simple aggregation such as simple or weighted arithmetic averages) was performed at this tier. Then, the result was sent to the medium tier (Fog node). This aspect reduces the data flow in the sensor tier, the network segment with less transmission capacity. The Fog node is located close to the IoT devices or along the communication path to the Cloud nodes. Fog node performs a complementary fusion of all data received by the sensor nodes using event processing techniques, thus allowing local decision making regarding the sensed area with low latency. It is essential to highlight that a Fog node has fewer resources than a Cloud node and just local knowledge, unlike Cloud nodes. Therefore, a Fog node can apply less sophisticated data fusion algorithms than those of a Cloud node. However, a Fog node can use more complex algorithms than an IoT device because it has a broader computational capacity to process and fuse information and receives a higher amount of data from different sources. Furthermore, if necessary, the Fog node can also directly act on the sensed area, aiming to reduce latency. Finally, the Fog node sends the data transformed by the data fusion and CEP methods to the high tier (Cloud node), which, according to the application, may be responsible for persistently storing the data and creating global inferences.

Our approach is also composed of geolocation and context-aware sensor nodes. In this way, when a new sensor node connects to the network, it is associated with a specific Fog based on its location and context information. For this purpose, the nodes start sending a broadcast message to the Fog nodes which includes its coordinates (latitude and longitude) and the type of data collected. If the Fog node is compatible with a datum type, it calculates the Euclidean distance between them and sends the data to the Cloud node. For better comprehension, we exemplify a sensor that sends temperature data to only connect to a Fog node that processes temperature data. The Cloud node checks the Fog with the lowest distance calculated and returns this information to the corresponding Fog node. In turn, the Fog node forwards the information to the sensor node. Then, the sensor node only sends the collected data to the corresponding Fog, so it is processed according to the approach requests.

Our proposal uses the CEP model of rules presented in [20] and according to the following mechanisms:

1. A simple event is extracted from the result of the simple fusion of the collected raw data; the aggregation was performed on a number N of sensor readings;
2. In the Fog tier, a rule mechanism describes the events generated from the received value. The aggregation performed at the IoT device tier can trigger other different events based on the obtained result. An example in the agriculture domain would be a temperature change. If the value has not changed since the last data were sent, then an event that results in a message being sent should be triggered; if changed, the calculation of a daily irrigation event should be triggered;
3. The set of simple events is aggregated, generating more complex events, and the Fog processes the event according to the defined rules;
4. Depending on the event complexity, whenever an event requires a global view, a new event sends the data to be processed in the Cloud.

**Figure 2:**
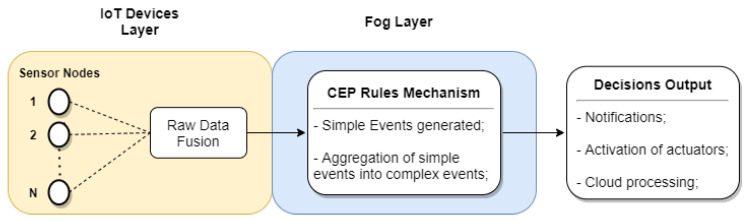
Example of the multi-tier CEP mechanism.

According to [21], these four mechanisms can be denominated: data receiver, rule mechanism, event executor, and event forwarder. We highlight that the processing of several simple events is complex event processing. Figure 2 exemplifies the multi-tier CEP mechanism.

Sensor nodes are associated with a specific Fog node according to their characteristics, such as location and context. For this connection, when a sensor node is added to the network, it sends a broadcast message to the Fog nodes, including coordinates’ parameters (latitude and longitude values) and the type of data collected. If the Fog node is compatible (same type), it calculates the Euclidean distance between them and sends its calculated value to the Cloud node. This is necessary because the Cloud is the only tier with a global knowledge of the network since the Fog nodes do not communicate with each other. The data compatibility is related to the data type that the Fog can process. For example, an IoT device sending temperature data will only connect to a Fog node that processes temperature data. The Cloud node verifies the nearest Fog based on the lowest calculated distance value and returns this information to the corresponding Fog node. The Fog node, in turn, forwards this information to the IoT device.

Using the CEP mechanism, we can separate the process of knowledge generation into partial processing and decision making. Thus, not only processing but network latency was also reduced. For example, when detecting an “abnormal” temperature, the sensor only sends the data to the decision-making part, which handles tasks of semantic reasoning, such as correlating the data received. In general, the CEP technique can be seen as a method that receives and correlates a set of simple events generating complex events. Thus, it is responsible for detecting simple events, using a cycle of filtering, correlation, contextualization, and data analysis from different sources.

### 3.1. Description of Application Rules and Events: Precision Agriculture

In the agricultural sector, achieving maximum crop yield at minimum cost is a production goal. In this context, decision-making is complex, as several factors affect the entire process, aiming to save water resources, mainly on irrigation.

The study of water in the soil is of great interest to agriculture because it affects the development and production of crops. A developing plant must supply the atmosphere’s demand for water through the amount it can extract from the soil. Soil water storage is reduced by evapotranspiration and replaced by precipitation or irrigation. Thus, soil water storage and availability in crop production are valuable, and characterizing the soil properties responsible for water retention is crucial. The soil moisture is estimated using three tensiometers at different soil depths in each monitoring point, which is used to estimate the soil matric potential. The matric potential is directly linked to moisture; the more humid the soil, the greater its potential [22].

Irrigation helps reserve enough water for the crop in its development stage. Its matric potential is used in irrigation management to determine when to irrigate, the amount of water, and the type of irrigation. The water distribution is on-demand, i.e., when water is always available by the irrigation project or pumped by the user and used when the crop needs.

The irrigation management determines irrigation timing by monitoring the soil matric potential (ψ) containing the highest concentration of crop roots (usually in the most superficial soil tier, monitored by the tensiometer). Another way to determine the time to irrigate is through the critical moisture $\theta_{c_{r}}$, also called ideal moisture for irrigation. This parameter is the soil water content from which the crop yield starts to be reduced, also having the possibility of evapotranspiration reduction (water consumption by cultivation). $\theta_{c_{r}}$ is only needed for the most superficial soil tier. The evapotranspiration measures the water consumption by crops, according to the developmental stage of the crop and weather conditions [22].

Another important variable to be considered in view of the plant’s needs is the irrigation water need (IWN). The IWN consists of the water provided entirely by irrigation to compensate for the water loss due to evaporation. It is obtained through a calculation considering the rainfall, water consumption, soil moisture at the crop root zone and the efficiency of the irrigation system placed in the field [22].

Similarly to the works in [23,24], we can also detect anomalies in sensor measures by comparing with measures from nearby sensors, in order to verify whether there was a sensor error, whether there is any water leakage, or whether, for some reason, the soil is too dry.

Based on these concepts, we define the rules and events of the case study presented in the following sections.

### 3.2. Rules and Events

The rules defined to manage the events are divided into two sets: monitoring rules and critical state rules. Each rule consists of a set of events that are executed according to its pattern and transformation primitives. These events are allocated to the tiers according to the capacity of the devices so that the approach performance is not affected. Thus, they directly influence the process of decision making by the user and also automatically according to the system’s behavior.

#### 3.2.1. Monitoring Rules

The first set of events of the monitoring rule are based on the primitives of filtration, aggregation, and projection. They are performed at the sensor tier. The projection is responsible for creating complex events (processed in the Fog tier) using a subset of its attributes, generated by aggregation (average of values obtained by the sensors) and by filtering (obtaining the maximum and minimum values). The rule of this first set was composed of the following events:

- EV1 (Simple): low matric potential alert;
- EV2 (Simple): high matric potential alert;
- EV3 (Simple): daily matric potential sending alert + sensor ID.

The second set is formed by simple and compound events based on the primitives of aggregation, enrichment, and composition performed in the Fog tier. Enrichment is the conference of the data necessary for calculations. Composition is the computations made from the data consulted. The events that compose this rule are:

- EV4 (Complex): matric potential variation;
- EV5 (Complex): daily irrigation water needed (IWN);
- EV6 (Complex): daily irrigation frequency verification.

The outputs of these events are messages displayed and sent to the user.

#### 3.2.2. Critical State Rules

The critical state rule is composed of events based on the primitives of enrichment, composition, negation and sequence performed at the Fog tier. For the use of the sequence primitive, an input control is necessary, which can be done through a time window, using a timestamp, or context, using an event identifier. This rule consists of the following events:

- EV7 (Complex): critical soil moisture (ideal for irrigation);
- EV8 (Complex): maximum water deficit.

These events result in automatic actions in the monitored area.

### 3.3. CEP Architecture

**Figure 3:**
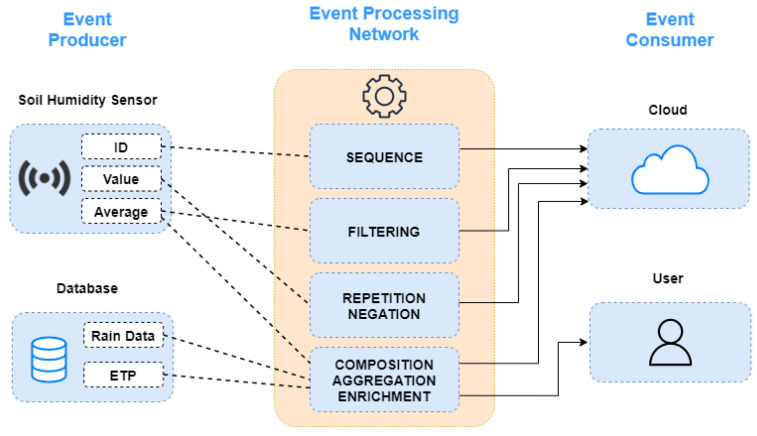
CEP architecture and primitives.

**Figure 4:**
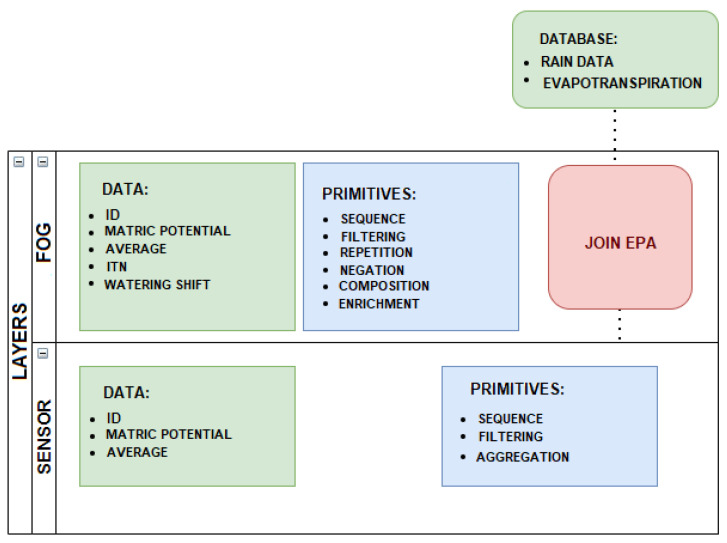
CEP primitives CEP according with tiers.

The proposed complex events processing (CEP) model is part of the data fusion mechanism to treat, analyze, and react to data flows as events. Event flows are the primary sources of input of the CEP model. The events are created through producers (sensors, users) that provide the data. The flow of events is processed through the CEP event processing agent, known as EPA. Specifically, the EPA follows the subsequent steps: reacts to events, analyzes, and manipulates them according to defined rules, and, if necessary, generates derivative (complex) events for the consumers. The EPA acts according to the primitives defined in each example, as illustrated in Figure 3 and Figure 4.

A CEP inference engine allows instantiating the presented concepts. In addition, it provides operations to define the types of events (schema and payload) and primitives in real-time to express continuous queries (PAEs) and interconnect them. The inference engines of the events are shown in Section 3.3.1.

#### 3.3.1. Description of CEP Rules and Events

We show the event inference engine applied to precision agriculture below.

**EV1:** Low Matric Potential (ψ)

**R1:** Matric Potential ≤ 55 kPa

- Send ψ, ID and sensor zone to the Fog tier
- Trigger EV4

**EV2:** High Matric Potential (ψ)

**R2:** Matric Potential ≥ 100 kPa

- Send ψ, ID and sensor zone to the Fog tier
- Trigger EV4

**EV3:** Daily Matric Potential (ψ)

**R3:** Number of readings (n) = 12

- Average the ψ
- Send the average ψ value and sensor ID to the Fog tier
- In the Fog tier, consult the daily precipitation value
- Store in variable p
- Consult Evapotranspiration value in the Database
- Store in variable Et0
- Trigger EV5

**EV4:** Matric Potential (ψ) Boundary

**R4:** If Matric Potential ≤ 55 kPa OR Matric Potential ≥ 100 kPa

**R4.1:** If Matric Potential ≤ 55 kPa

- See the (ψ) of sensors with different IDs from the one that generated the event in the same zone

**R4.1.1:** If 60 kPa < ψ ≤ 100 kPa

- Send message to user to check possible sensor malfunction

**R4.1.2:** If ψ ≤ 55 kPa

- Trigger EV8

**R4.2:** If Matric Potential ≥ 100 kPa

- See the (ψ) of sensors with different IDs from the one that generated the event in the same zone

**R4.2.1:** If 60 kPa < ψ ≤ 100 kPa

- Send message to user to check possible sensor malfunction

**R4.2.2:** If ψ ≥ 100 kPa

- Verify the precipitation

**R4.2.3:** If p = null

- Send message to user to check water leak

**EV5:** Daily Irrigation Water Needed (IWN)

**R5:** If R3 was activated

- Calculate IWN
- Send message with IWN to the user
- Trigger EV6

**EV6:** Irrigation Frequency Definition

**R6:** If R4 was activated

- Calculate and schedule irrigation time
- Send message with irrigation time to the user

**EV7:** Critical Soil Moisture Alert

**R7:** IWN ≤ Critical soil moisture (60kPa)

- Activate zone irrigation
- Send message to the user

**EV8:** Maximum Water Deficit

**R8:** If R4.1.2 was activated

- Activate zone irrigation
- Send message to the user

We performed the auditing as a data processing and decision-making tool to complement this set of rules and events. The event-processing programming differs from regular programming and thus needs its own auditing tool. Auditing is the ability to investigate whether processes have been properly applied. The investigation may refer to whether a process complies with external regulations or internal policies or whether a decision has been appropriately made.

#### 3.3.2. Auditing

Given the rules and events presented in Section 3.3.1, we performed the auditing of the CEP model using a static analysis of the network. The auditing process for a CEP model is based on the event log, a list that retains the raw and derived events performed in the given processing. The audit was made based on consultations in this event log. The query is similar to the dynamic analysis process of the validation process, as it depends on the amount of information from the execution of a given event.

For the auditing of the CEP model, two primary consultations were performed based on the concept presented in [25]: (i) consult all antecedents of a given event or activation of an EPA instance (rule); and (ii) consult all the consequences of a given event or activation of an EPA instance (rule).

We can observe from these two main consultations that the objective of the audit is to check the background and consequences of a given processed event, i.e., to check the background of the finalizing events and the consequences of the initial events. Thus, for the model applied to the approach proposed in this article, it is necessary to audit the following events:

- Consult 1: EV4, EV6, EV7, and EV8.
- Consult 2: EV1, EV2, and EV3.

**Figure 5:**
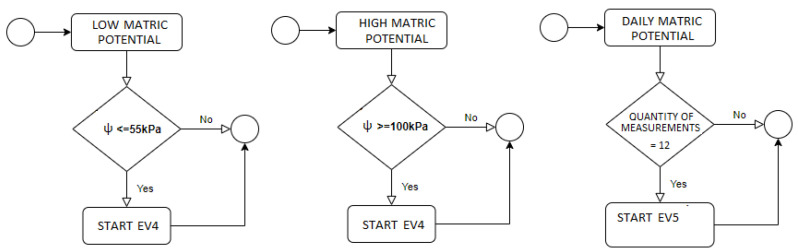
Log of events 1, 2, and 3.

**Figure 6:**
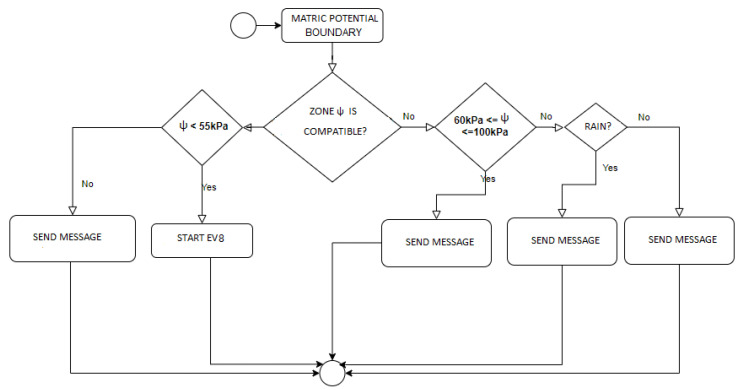
Log of event 4.

**Figure 7:**
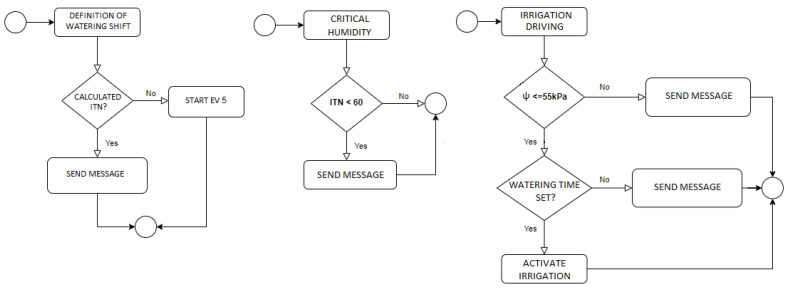
Logs of events 6, 7, and 8.

Figure 5, Figure 6 and Figure 7 present the logs of the consulted events. From the logs, we can verify that event processing is meeting the application’s needs. Exemplifying the Event 1 log (Figure 5), the expected performance according to the low matric potential is irrigation unless a sensor malfunction has occurred. Then, following the processing of event 1, it ends by triggering event 4 in case the rule is activated. Now, observing the log presented in Figure 6, we verified that this event ends by exactly meeting the need to trigger event 1: or ends by sending a message to the user informing of the sensor malfunction, or sending a message to trigger manual irrigation, or, finally, triggering event 8. Event 10 checks the activation of the EPA instance, testing the rule, and ends with the triggering of the automatic irrigation or requesting that the same be done manually, as we can see in the log of Figure 7.

Thus, based on any consult executed in the logs presented, the application’s need is satisfied at the end of the processing—starting from event 2 (Figure 5) and ending at event 4 (Figure 6), etc. In this way, the audit of the CEP model used in the proposed approach was successfully satisfied.

## 4. Materials and Methods

This section presents the performed experiments. We divided them into two experimental groups, with different goals. The first group refers to the experiments performed to analyze the proposal’s performance using the metrics of accuracy, precision, recall, F1-Score, data traffic, and time to send packages. The second group refers to the implementation of the rules responsible for controlling the irrigation process, the validation of the soil moisture management, and the reaction time of the actuators to verify the response time and the CEP efficiency. Table 2 summarizes the experiments, objectives, and metrics.

### 4.1. Experiment I: Performance Evaluation

This section presents the first performance evaluation, executed to verify the quality of the decision generated and the performance in terms of data traffic and average sending time with the application of the CEP approach. Both aspects are critical to assess the performance of the large-scale deployment of wireless sensors. To create a proof of concept (PoC) of our proposal, we used a real coconut field of Embrapa Agroindustrial [26] as a model to simulate an environment for our experiments. The experimental field is located in Paraipaba-Ce.

#### Experiment I: Design

Our simulated field consists of 25 coconut trees, in which temperature sensors monitor fifteen trees, and soil moisture sensors monitor ten trees. In addition, five Fog nodes communicating with the Cloud were implemented. Furthermore, we simulated two other scenarios to verify the approach’s scalability by varying the number of nodes as follows: (i) 50 sensor nodes (25 temperature, 25 soil moisture) and 10 Fog nodes; and (ii) 100 sensor nodes (50 temperature, 50 soil moisture) and 20 Fog nodes.

We used a discrete event simulator focused on, but not restricted to, Fog environments, called Yet Another Fog Simulator (YAFS) to run the simulations. YAFS was built to analyze the application’s design and it incorporates strategies for positioning, programming, and routing. YAFS has similar characteristics to the iFogSim simulator and uses it as a reference [27]. According to [28], iFogSim is one of the most widely used Fog simulators for scenario simulations using Fog and Cloud computing. YAFS includes more functionality than current simulators for modeling IoT scenarios and is easier to add extensions. We highlight the following points of YAFS:

- It provides a network vision that allows the modeling of the communication links among machines, users and end-devices;
- Each workload source represents the connection of a user or an IoT sensor or actuator that demands a service and can be created, changed or dynamically removed, enabling the modeling of the user movements in an ecosystem;
- Custom processes can be invoked at runtime to provide flexible implementations of real events;
- It provides post-simulation data analysis based on two types of events: workload generation and computation, and link transmissions.

The sensor readings were carried out over a 20-min time frame so that we capture the hourly average. Every twelve hours, the sensor node performs a simple aggregation function, calculating the arithmetic mean. Then, each sensor sends the value to its respective Fog node. Then, the Fog node transforms the received data into information using data fusion and CEP techniques such as filtering and composing complex events. CEP works as an inference engine, filtering, correlating, and composing new events based on the information obtained through the fusion of the data. For example, after consecutive readings of temperature values greater than 29 ºC, an event is created that exhibits a temperature alert message on display. Likewise, a new event is created to send an urgent message to prevent the soil from becoming too dry after displaying five messages without change in the monitored area (without action).

The sensor nodes have 16 MB RAM, while Fog nodes have 4 GB and Cloud nodes have 8 GB. These values were chosen to be realistic, closer to the device’s configuration often used. Thus, each sensor node is connected to a Fog node, while each Fog node is connected to the Cloud node. For simulation purposes, only one Cloud node was used in all scenarios. The parameters configured in the experiments performed are summarized in Table 3.

To better understanding the metrics of quality decisions, the basic knowledge of each concept is necessary. False positives and false negatives are illustrated utilizing the confusion matrix, a table indicating the errors and successes of the evaluated model, comparing them with the expected result. In this matrix, the following values are shown:

- True positive (TP): values that correspond to the positive of the value read; in the case of the architecture presented, the value must be within the normal temperature range (up to 29 ºC) and acceptable humidity (greater than or equal to 60 KPa);
- False negative (FN): error in which the model predicted a negative result (that is, outside the normal temperature range or acceptable soil moisture) when the real value was within the positive range (described above);
- False positive (FP): error in which the model predicted a positive result when the real value was within the negative range (that is, temperature > 29 ${}^{\circ }$C or soil moisture < 60 KPa);
- True negative (TN): correct classification of values within the high temperature range (greater than 29 ${}^{\circ }$C) or dry soil (less than 60 KPa) before and after the data fusion is applied.

When all the terms (TP, FN, FP, and TN) are computed, resulting in the confusion matrices, it is possible to calculate the following metrics for assessing the quality of the data: accuracy (Ac); precision (P); recall (R); and F1-Score (F1) using the Equations (1)–(4):

(1) $$Ac=\frac{TP+TN}{TP+FP+TN+FN}$$

(2) $$P=\frac{TP}{TP+FP}$$

(3) $$R=\frac{TP}{TP+FN}$$

(4) $$F1=2\times \frac{P\times R}{P+R}$$

Regarding these metrics, accuracy provides a general analysis of the model considering all the data collected; this metric indicates the percentage of data that were correctly classified. The precision metric provides a percentage of how many positive instances were correctly ranked, while the Recall metric was used to indicate the relationship between positive predictions performed correctly and all data that are actually positive. The F1-score metric indicates the overall behavior of accuracy and recall. If the F1-score is low, it is understood that one of the two metrics is low. Regarding this last metric, it is important to highlight the harmonic mean of accuracy and recall. When the values are close, the result is similar to the arithmetic mean [29].

### 4.2. Experiment 2: Validation of CEP Model Implementation and Reaction Time

This section presents the implementation of rules directly linked to irrigation control and the validation of its effectiveness using a program that simulates the sensors and the soil matric potential values in real time.

#### Experiment 2: Design

**Figure 8:**
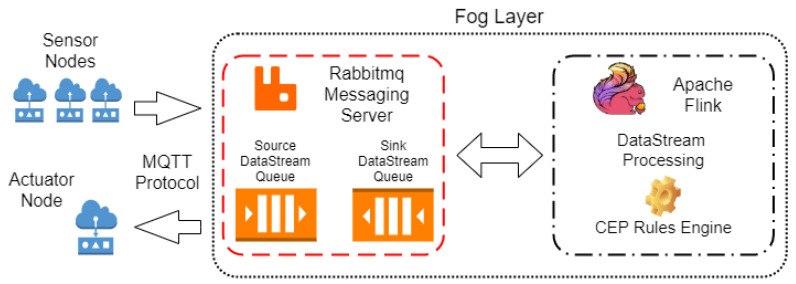
Experiment 2: implementation of Fog tier components.

Figure 8 shows the components implemented for Experiment 2, where sensors and actuators nodes exchange information with the Fog tier. We used three soil moisture sensors and an actuator that controls irrigation in the zone in the field. We used Python to simulate both components, with soil conditions and moisture progression according to the irrigation state (on or off). The Fog tier comprises two components: a Rabbitmq messaging server containing two queues (data input and output) and a module dedicated to the data stream and complex event processing (CEP), which was implemented using Apache Flink the FlinkCEP library. Communication between the Fog tier and the IoT nodes in the field was through the MQTT protocol. Rabbitmq was chosen as the messaging service because the tool provides a native plugin that supports MQTT messages.

In order to validate the effectiveness of the rules implemented in Apache Flink in controlling irrigation, we programmed the sensors to send the matric potential values every minute to the Fog tier. Upon detecting event 9 or 10, the irrigation was turned on. We used the value of 20 kPa as a reference for the ideal value of matric potential as a trigger to turn off the irrigation. We obtained this information from experts in the agricultural area.

## 5. Results

### 5.1. Experiment I: Results and Discussion

This section presents the results of the performance evaluation experiment concerning the following metrics: false positives, false negatives, accuracy, precision, recall, F1-Score, and data traffic. We use three scenarios as described below.

#### 5.1.1. Scenario A: 25 Sensor Nodes, 5 Fog Nodes

From the simulation of scenario A, we generate the confusion matrix, presented in Table 4. The matrix presents the results regarding VP, FP, FN, and VN from the comparison of the raw soil moisture data (generated in the sensors) to the final information provided by our fusion proposal. In total, during the simulations performed, soil moisture sensors generate 360 readings.

Table 5 refers to the confusion matrix generated from the 540 temperature data originated in the sensor nodes. Similar to the soil moisture data, we compared the raw temperature data to the information generated after applying the techniques. Based on the values found in the confusion matrices (Table 4 and Table 5), the other metrics were calculated and shown in Table 6.

According to the results, our CEP approach presented 100% accuracy in the soil moisture data and approximately 99% in the temperature data. Accuracy provides information about how much the false positive influences the information generated: the percentage of correctness when the soil has a moisture and temperature considered adequate for the plant, thus preventing the plant from being without its proper irrigation and reducing its productivity. Thus, the CEP approach presented a precision of 100% for the two observed data sets. Recall informs how the false-negative has affected the results obtained. In our scenario, the recall informs the amount of correctness regarding the need to use water. The approach presented a recall above 99% for the observed data, which prevents the plant from being unnecessarily irrigated, thus saving water. The F1-Score is a harmonic mean that verifies the disparity between precision and recall, supplying greater credibility to the observed values. This metric checks whether the accuracy or recall is well below the expected value. The CEP approach obtained an F1-score of 99%, thus showing that in all aspects, our CEP approach provided very coherent information when compared to the raw values generated by the sensors.

**Figure 9:**
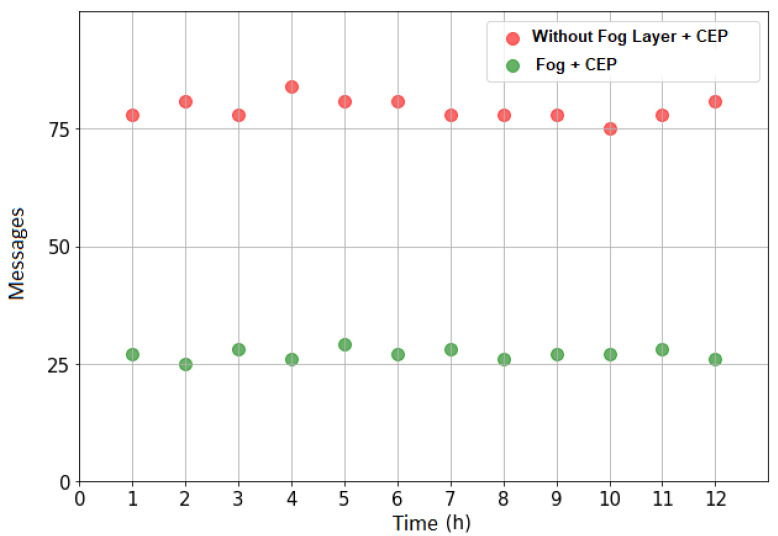
Data traffic—scenario A.

Figure 9 shows the data traffic with and without the Fog tier and the processing of complex events. It can be seen that the traffic data decreases with the complex data processing in the Fog tier.

In the subsequent scenarios, the scalability of the CEP approach is checked for scenarios with a higher density of nodes.

#### 5.1.2. Scenario B: 50 Sensor Nodes, 10 Fog Nodes

In scenario B, 1800 raw data were generated, with 900 for soil moisture and 900 for temperature. The confusion matrices presented in Table 7 and Table 8 were obtained from comparing the raw data with the final information generated using the proposed approach.

Based on the data from the confusion matrices, we calculated accuracy, precision, recall, and F1-Score. As shown in Table 9, all parameters remain above 99% when the network has an average density of nodes. Thus, we can say that the CEP approach remains reliable when applied to a network with average node density.

**Figure 10:**
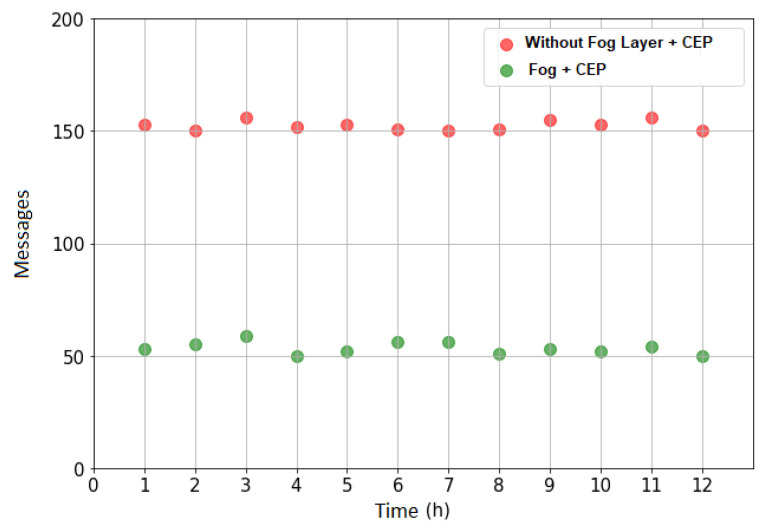
Data traffic—scenario B.

Figure 10 shows the data traffic with and without the Fog tier and the processing of complex events in a higher density scenario. It can be seen that the traffic data also decreases with the complex data processing in the Fog tier.

#### 5.1.3. Scenario C: 100 Sensor Nodes, 20 Fog Nodes

The experiment in scenario C verifies the approach’s behavior in a network with a more significant number of nodes. Thus, to validate the scalability of the proposal, 3600 raw data were generated: 1800 soil moisture and 1800 temperatures. The confusion matrix (Table 10) was obtained by collecting the soil moisture on the field using the proposed approach. The matrix (Table 11) shows the true and false positives and negatives of the measured temperature collected on the field using CEP and fusion techniques.

Based on the data from the confusion matrices (Table 10 and Table 11), we calculated the accuracy, precision, recall, and F1-Score. As shown in Table 12, all parameters remain above 99% when the network has a high node density. Thus, we can conclude that the approach remains reliable when applied to a network with high node density.

**Figure 11:**
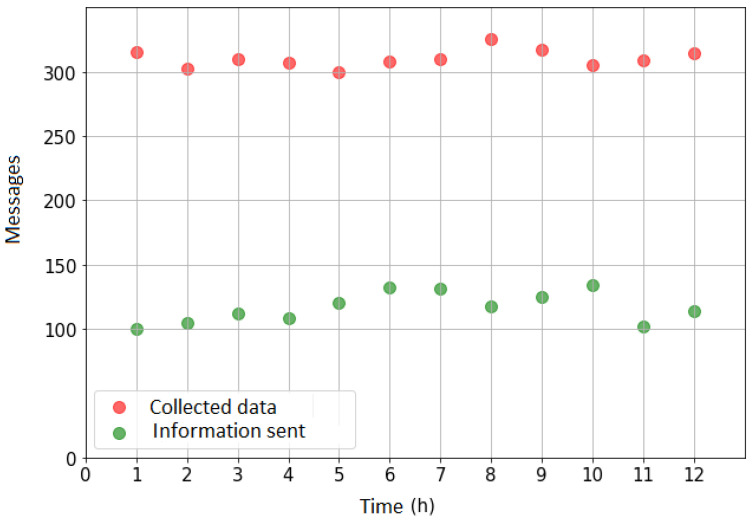
Data traffic—scenario C.

Observing the data traffic in the simulated scenario (Figure 11), it is possible to verify an average reduction of 82% when compared the use of CEP and fusion techniques to the flow of the network without the use of the techniques.

**Figure 12:**
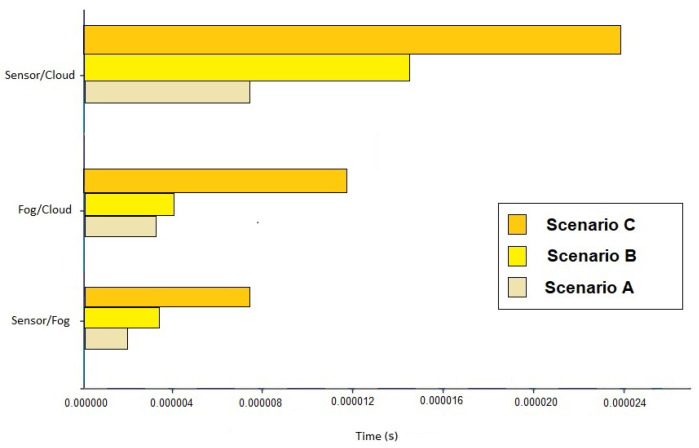
Average message sending time in the 3 scenarios.

Another metric to be analyzed is the average time to send a message to its final destination. By processing data obtained by the fusion and the processing of events, some data that would be initially sent to the Cloud are treated and resolved in the previous tiers. Our results (Figure 12) show that the average message sending time is 77% shorter when processing is performed in the Fog tier without sending all data to the Cloud.

### 5.2. Experiment 2: Results and Discussion

**Figure 13:**
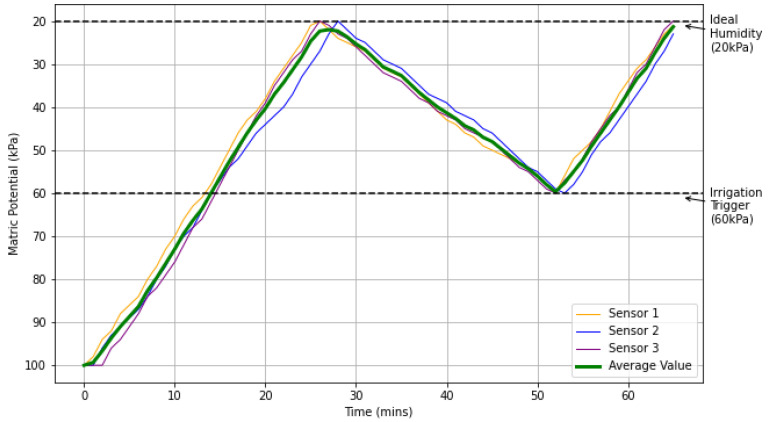
Matric potential (soil moisture) control.

Figure 13 shows the behavior of the matric potential values during approximately sixty minutes of simulation. According to Figure 13, we can conclude that the objective of control in a simulated environment was achieved since the value of the matric potential remained within the defined limits.

**Figure 14:**
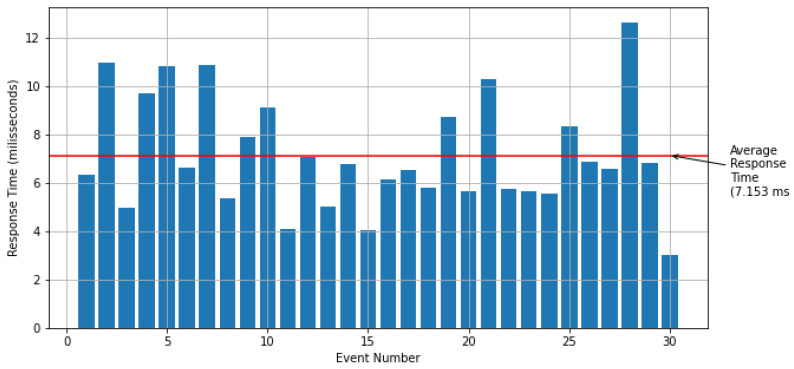
Actuator response time.

The second part of the experiment evaluated the response time of the irrigation activation in the actuator node by identifying the event in the CEP engine. The response time of thirty events was analyzed. Figure 14 shows the test results. The average response time obtained was approximately 7.15 ms on a local network.

## 6. Conclusions

We presented a multi-tier hierarchical CEP approach in Fog focused on the smart agriculture domain. Our approach is geolocation and context-aware, in which the new sensor nodes in a network connect to a Fog node based on the data type and the Euclidean distance between them. To evaluate the proposal, we performed simulations in different scenarios with different network densities and configurations. The experiments achieved promising results, such as accuracy, precision, recall, and F1-Score which were consistently above 99%. Thus, we can claim that the information obtained using the proposed approach is reliable and consistent with the reality of the monitored environment. In order to validate the effectiveness of the CEP engine (rules and events), we conducted tests using an irrigation scenario and it was successfully completed.

Our results show that the objective of control in a simulated environment was achieved since the value of the matric potential remains within the defined limits. The second part of the experiment evaluated the response time of the irrigation activation in the actuator node by identifying the event in the CEP engine. The average response time obtained was approximately 7.15 milliseconds on a local network, showing that the CEP engine meets the application’s needs.

In future work, we intend to investigate the self-configuration of the nodes regarding the best characteristics to be considered for the choice of the Fog node, such as the processing power. Another point to be explored in future work is the use of the data stream-oriented CEP mechanism, which is similar to SQL, instead of rule-oriented modeling.

## Figures and Tables

**Table 1:** Related work.

Article	Tiers	Organization	CEP	Data Fusion	Implementation, Simulation or None	Cloud, Fog or None
*Our approach*	*3*	*Hierarchical*	*Yes*	*Yes*	*Simulated*	*Fog*
[7]	2	Hierarchical	Yes	No	Implemented	Fog
[8]	2	Hierarchical	No	Yes	Implemented	Fog
[9]	3	Hierarchical	No	Yes	Implemented	Fog
[10]	2	Distributed	Yes	Yes	Implemented	Fog
[11]	2	Collaborative	Yes	No	Implemented	Fog
[12]	1	Distributed	Yes	No	Simulated	Fog
[13]	1	Distributed	Yes	No	Simulated	Fog
[14]	3	Hierarchical	No	No	Implemented	Fog
[15]	3	Distributed	No	No	Implemented	Fog
[16]	3	Distributed	No	No	Simulated	Fog/Edge
[17]	3	Hierarchical	No	No	None	Fog
[18]	3	Distributed	No	No	Simulated	Fog

**Table 2:** Experiments.

Experiment	Objectives	Metrics
**I**	Performance Evaluation	Accuracy, Precision, Recall,
		F1-Score e Data Traffic
**II**	Implementation Validation and	CEP Efficiency and Response Time
	Reaction Time	

**Table 3:** Experiments.

Experiment	Scenario	Evaluation
**1**	25 Sensor Nodes	Accuracy, Precision, Recall,
	5 Fog Nodes	F1-Score and Data Traffic
**2**	50 Sensor Nodes	Scalability
	10 Fog Nodes	
**3**	100 Sensor Nodes	Scalability
	20 Fog Nodes	

**Table 4:** Confusion matrix—soil moisture.

	Acceptable Moisture	Dry Soil
**Acceptable Moisture**	291	0
	True Positive	False Positive
**Dry Soil**	0	69
	False Negative	True Negative

**Table 5:** Confusion matrix—temperature.

	Normal Temperature	High Temperature
**Normal Temperature**	429	0
	True Positive	False Positive
**High Temperature**	3	108
	False Negative	True Negative

**Table 6:** Evaluation metrics—scenario 1.

	Soil Moisture	Temperature
**Accuracy**	1	0.994
**Precision**	1	1
**Recall**	1	0.993
**F1-Score**	1	0.993

**Table 7:** Confusion matrix—soil moisture.

	Acceptable Soil Moisture	Dry Soil
**Acceptable Soil Moisture**	767	4
	True Positive	False Positive
**Dry Soil**	3	126
	False Negative	True Negative

**Table 8:** Confusion matrix—temperature.

	Normal Temperature	High Temperature
**Normal Temperature**	750	3
	True Positive	False Positive
**High Temperature**	4	143
	False Negative	True Negative

**Table 9:** Evaluation metrics—scenario B.

	Soil Moisture	Temperature
**Accuracy**	0.992	0.992
**Precision**	0.995	0.996
**Recall**	0.996	0.995
**F1-Score**	0.995	0.995

**Table 10:** Confusion matrix—soil moisture.

	Acceptable Moisture	Dry Soil
**Acceptable Moisture**	1616	7
	True Positive	False Positive
**Dry Soil**	6	171
	False Negative	True Negative

**Table 11:** Confusion matrix—temperature.

	Normal Temperature	High Temperature
**Normal Temperature**	1604	4
	True Positive	False Positive
**High Temperature**	8	184
	False Negative	True Negative

**Table 12:** Evaluation metrics—scenario C.

	Soil Moisture	Temperature
**Accuracy**	0.992	0.993
**Precision**	0.996	0.997
**Recall**	0.995	0.995
**F1-Score**	0.995	0.996
